# Surveillance of low pathogenic novel H7N9 avian influenza in commercial poultry barns: detection of outbreaks and estimation of virus introduction time

**DOI:** 10.1186/1471-2334-14-427

**Published:** 2014-08-01

**Authors:** Amy Pinsent, Isobel M Blake, Michael T White, Steven Riley

**Affiliations:** MRC Centre for Outbreak Analysis and Modelling, Department of Infectious Disease Epidemiology, Imperial College London, Norfolk Place, London, UK

**Keywords:** H7N9, Influenza, Surveillance, *R*_0_, Poultry

## Abstract

**Background:**

Both high and low pathogenic subtype A avian influenza remain ongoing threats to the commercial poultry industry globally. The emergence of a novel low pathogenic H7N9 lineage in China presents itself as a new concern to both human and animal health and may necessitate additional surveillance in commercial poultry operations in affected regions.

**Methods:**

Sampling data was simulated using a mechanistic model of H7N9 influenza transmission within commercial poultry barns together with a stochastic observation process. Parameters were estimated using maximum likelihood. We assessed the probability of detecting an outbreak at time of slaughter using both real-time polymerase chain reaction (rt-PCR) and a hemagglutinin inhibition assay (HI assay) before considering more intense sampling prior to slaughter. The day of virus introduction and *R*_0_ were estimated jointly from weekly flock sampling data. For scenarios where *R*_0_ was known, we estimated the day of virus introduction into a barn under different sampling frequencies.

**Results:**

If birds were tested at time of slaughter, there was a higher probability of detecting evidence of an outbreak using an HI assay compared to rt-PCR, except when the virus was introduced <2 weeks before time of slaughter. Prior to the initial detection of infection *N*_*sample*_ = 50 (1%) of birds were sampled on a weekly basis once, but after infection was detected, *N*_*sample*_ = 2000 birds (40%) were sampled to estimate both parameters. We accurately estimated the day of virus introduction in isolation with weekly and 2-weekly sampling.

**Conclusions:**

A strong sampling effort would be required to infer both the day of virus introduction and *R*_0_. Such a sampling effort would not be required to estimate the day of virus introduction alone once *R*_0_ was known, and sampling *N*_*sample*_ = 50 of birds in the flock on a weekly or 2 weekly basis would be sufficient.

**Electronic supplementary material:**

The online version of this article (doi:10.1186/1471-2334-14-427) contains supplementary material, which is available to authorized users.

## Background

Outbreaks of avian influenza (AI) remain an ongoing threat to the commercial poultry industry globally. Such outbreaks can result in substantial economic losses, due to the cost of implementing control measures and the financial loss associated with culling infected flocks [1]. In the Netherlands a highly pathogenic AI (HPAI) outbreak in 2003 led to the culling of 30 million animals resulting in direct costs of 250 million euros [2]. In the United States (US) outbreaks of low pathogenic AI (LPAI), once detected can also cost millions of dollars to control and contain [3–5].

Birds infected with LPAI subtypes (e.g. H9N2, H6N2, H7N9) often present with few or no symptoms, making detection of LPAI particularly challenging. Although LPAI does not present itself as a serious concern to animal health, LPAI subtypes H5 and H7 have been shown to evolve by mutation into highly pathogenic avian influenza (HPAI) [6–8]. Therefore higher numbers of LPAI cases increase the chance that mutations associated with HPAI will arise. Whilst outbreaks of AI in commercial poultry production systems in the developed world are infrequent, an average of two to three cases of H5/H7 are reported in the US annually, in addition to other LPAI subtypes, hence AI outbreaks remain an on-going threat to the commercial poultry production industry.

The emergence of a novel H7N9 LPAI subtype in China [9] presents itself as a serious concern for human health in addition to the risks to the commercial poultry industry. Over 400 human infections have been reported in China, as of June 2014 [10] with a case fatality ratio upon admission to hospital for humans estimated to be 32-36% [11, 12] for the first wave of infections, with estimates from the second wave also within this range [13]. However, infected birds are not reported to have exhibited symptoms [14]. The continuous infection events (spill-over) from birds to humans increase the chance that reassortment with human endemic viruses will occur, or that human adaptation mutations will become fixed. Since the detection of this virus a number of subsequent reassortment events have been reported [15, 16], suggesting that this novel lineage may display a high propensity to reassort. Therefore, identification and control of H7N9 infection within the commercial poultry sector is of paramount importance for human and animal health.

There are currently believed to be at least 17 billion commercial chickens worldwide [17], providing a very large susceptible population for AI subtypes to circulate in. Therefore regular and long term surveillance is strongly encouraged and is the most effective way of controlling and identifying AI in poultry [18]. One important aspect of this control policy is the systematic surveillance and diagnosis of infection [19]. Two tests are commonly used to identify whether birds have been infected: real-time polymerase chain reaction (rt-PCR) [20] and the hemagglutinin inhibition assay (HI). rt-PCR identifies the causative agent of infection and detects current infection by detecting the presence of viral RNA. HI assays test for the presence of antibodies to the hemagglutinin (HA) antigen [21], and hence detect evidence of past infection.

It is unfeasible to test all birds within a barn on a daily basis; therefore optimal sampling strategies need to be considered to detect evidence of a LPAI outbreak and infer parameters of epidemiological interest. Mechanistic models can be used to successfully infer epidemiological events of interest [5], and thus improve surveillance and enable effective response strategies in the event of an outbreak. Models can be used to infer important events such as the day of the virus introduction in an outbreak setting, which is crucial for preventing onwards transmission of infection and identifying the source of infection. For example, Bos *et al*[22] used mortality data from a HPAI outbreak of H7N7 in the Netherlands to estimate the day of virus introduction into flocks. However given mortality does not occur due to infection during LPAI outbreaks, new methodology is required.

The ability to detect infection within a flock depends on a number of factors including; the type of test used to identify infection, the frequency of sampling, the day of virus introduction (*T*_*s*_) and the within-flock transmissibility of the strain, *R*_0_ (the average number of secondary cases generated by an infectious primary case). There is a complex interaction between these parameters, and we explore how they can alter the chance of detecting infection in a barn. We first evaluated the probability of detecting evidence of an avian influenza H7N9 outbreak at time of slaughter across a range of values of *R*_0_ and *T*_*s*_ using two different available tests. Secondly, we tested our ability to estimate *R*_0_ and *T*_*s*_ when sampling birds is performed on a weekly basis. Such estimation would be useful in the event of a novel outbreak where little was known about the pathogen. Lastly we investigated how different sampling frequencies affect the ability to correctly estimate the *T*_*s*_ into a barn under a range of different *R*_0_ values. Such inference would be useful if an outbreak was or had occurred in a neighbouring area (when it would be reasonable to assume *R*_0_ was already known). A deterministic mathematical model of H7N9 transmission with a stochastic observation process was used to investigate these three aims.

## Methods

### Mathematical model of LPAI transmission in a commercial poultry setting

**Figure 1:**
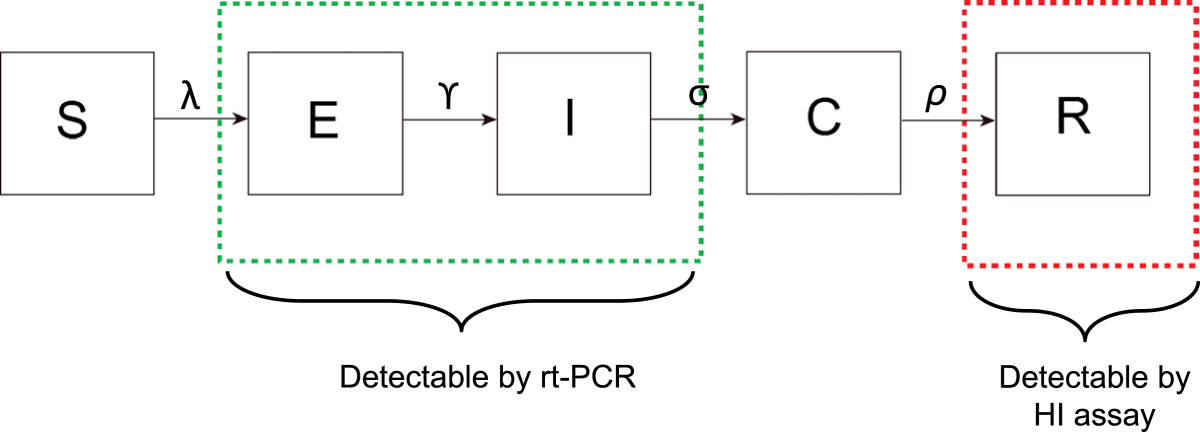
**Flow diagram of the SEIR model structure.** Boxes around compartments indicate which states will result in a positive test for the rt-PCR test and HI assay.

We developed an extension of the deterministic susceptible, exposed, infectious, recovered model (SEIR), with a time lag after completion of the infectious period before birds could be detected as recovered and sero-positive for infection; we called this the seroconverting class (*C*). A flow diagram of the system is illustrated in Figure 1. All parameter definitions and values are defined in Table 1.

**Table 1:** **Table of parameters used in the model**

Parameter	Description	Value
*R*_0_	Basic reproductive	3, 5, 7, 10
	number	
*γ*	Mean latent	2* [27] (Pantin-Jackwood,
	period (days)	personal communication)
1/(*γ*/n_*o*_)	Average duration spent	0.4
	in each compartment	
	during the latent	
	period (days)	
*σ*	Mean infectious	6* [27] (Pantin-Jackwood,
	period (days)	personal communication)
1/(*σ*/n_*o*_)	Average duration	1.2
	spent in each	
	compartment during	
	the infectious	
	period (days)	
*ρ*	Mean time to	8 [24]
	sero-conversion	
	post infection (days)	
1/(*ρ*/n_*c*_)	Average duration	0.8
	spent in each	
	compartment during	
	the sero-converting	
	period (days)	
Sensitivity (*ϕ*)	Sensitivity of	95% [20, 28, 29]
	rt-PCR test	
Specificity	Specificity of	100 % [20, 28, 29]
	rt-PCR test	
Sensitivity (*η*)	Sensitivity of	86% [30–32]
	HI assay	
Specificity	Specificity of	100 % [30–32]
	HI assay	
N	Population size	5000*
n_*o*_	Number of	5
	compartments for	
	exposed and	
	infectious periods	
n_*c*_	Number of seroconverting	10
	compartments	
Start time (*T*_*s*_)	Time infection	0, 7, 20, 40
	is introduced (days)	

Values of parameters that have an *next to them indicate uncertainty in these values, these values were varied in the sensitivity analysis.

A flock of N susceptible poultry are moved into the barn once every 8 weeks, this cohort of birds remain within the barn for the full 8 weeks until they are transported. Here we follow one cohort over the 8 week period, i.e. all birds enter and leave together. At the start all birds are assumed to be susceptible and previously unexposed to the virus. Birds experience a force of infection *λ*(t), which depends on the effective contact rate *β*, (i.e. the mean number of birds a bird comes into contact with and transmits to per unit time), and I (t), the total number of infectious birds in the barn at each time point. Therefore *λ*(t), the force of infection experienced by a susceptible bird in the barn per unit time can be written as:

1 λ(t)=βI(t)

Once a bird has come into contact with virus it is classified as exposed. Exposed birds are not infectious, and have a mean latent period *γ*. Both exposed and infectious birds are assumed to be asymptomatic. Birds have a mean asymptomatic infectious period *σ*, following which birds progress into a seroconverting class where they no longer transmit infection. The seroconverting class represents the time lag that occurs as antibodies are developed to infection prior to the birds being detectable as sero-positive and recovered. After this period birds become immune to re-infection with a mean duration *ρ* and move to the *R* class. We divide the exposed (*E*), infectious (*I*) and sero-converting (*C*) classes into 5, 5 and 10 compartments respectively to reduce the variance of the time spent in each compartment [23]. This ensures that the majority of birds progress through each class at a rate which ensures that under 10% of birds seroconvert 14 days post infection but that 90% have converted 21 days post infection [24].

The system is governed by the following series of differential equations:

2 $$\frac{\text{dS}}{\text{dt}}=-\mathrm{\lambda S}$$

3 $$\frac{{\text{dE}}_{1}}{\text{dt}}=\mathrm{\lambda S}-E_{1}\left(\frac{n_{o}}{\gamma }\right)$$

4 $$\frac{{\text{dE}}_{2:n_{o}}}{\text{dt}}=E_{1:(n_{o}-1)}\left(\frac{n_{o}}{\gamma }\right)-E_{2:n_{o}}\left(\frac{n_{o}}{\gamma }\right)$$

5 $$\frac{{\text{dI}}_{1}}{\text{dt}}=E_{n_{o}}\left(\frac{n_{o}}{\gamma }\right)-I_{1}\left(\frac{n_{o}}{\sigma }\right)$$

6 $$\frac{{\text{dI}}_{2:n}}{\text{dt}}=I_{1_{:}(n_{o}-1)}\left(\frac{n_{o}}{\sigma }\right)-I_{2:n_{o}}\left(\frac{n_{o}}{\sigma }\right)$$

7 $$\frac{{\text{dC}}_{1}}{\text{dt}}=I_{n_{o}}\left(\frac{n_{o}}{\sigma }\right)-C_{1}\left(\frac{n_{c}}{\rho }\right)$$

8 $$\frac{{\text{dC}}_{2:n_{c}}}{\text{dt}}=C_{1_{:}(n_{c}-1)}\left(\frac{n_{c}}{\rho }\right)-C_{2:n_{c}}\left(\frac{n_{c}}{\rho }\right)$$

9 $$\frac{\text{dR}}{\text{dt}}=C_{n_{c}}\left(\frac{n_{c}}{\rho }\right)$$

We infer the value of *R*_0_ from the model by estimating the transmission rate *β*, using the following relationship.

10 $$R_{0}=\beta \sigma (N-1)$$

### Testing for infection or antibodies

At a defined time point infection was introduced into the barn. This represents the external introduction of virus, either from contact with other infectious birds nearby or fomites.

We used the transmission model to simulate collected surveillance data. We assumed *N*_*sample*_ = 50 (1%) of birds, were sampled randomly from the barn at a given time point *(i)* and tested for infection or the presence of antibodies. The number of positive birds sampled at a given time point is a random variable from the following binomial distribution:

11 $$I_{\text{data}}∼B(N_{\text{sample}},P_{i})$$

*E* and *I* birds test positive for infection with an rt-PCR test, therefore the probability of identifying a positive bird in the sample is:

12 $$P_{i}=\frac{E_{i}+I_{i}}{N}\phi$$

where *ϕ* is the sensitivity of the rt-PCR test. Only *R* birds test positive for infection using an HI assay. Therefore the probability of identifying a previously infected bird in the sample is given by:

13 $$P_{i}=\frac{R_{i}}{N}\eta$$

where *η* is the sensitivity of the HI assay.

### Detecting evidence of infection at time of slaughter

We consider a surveillance system for detecting present or past infection at the time of slaughter. This requires detection of at least 1 bird in our *N*_*sample*_ = 50 sample, the probability can be written as follows:

14 $$1-{\left(1-P_{i}\right)}^{N_{\text{sample}}}$$

### Simulating sampling data

We chose to only consider testing scenarios using the rt-PCR test, as this test is more suitable for real-time detection of outbreaks than the HI assay.

For the first scenario to estimate *T*_*s*_ and *R*_0_ we simulated sampling data on a weekly basis using an rt-PCR test for four different introduction times: i) day 0, ii) day 7, iii) day 20, and iv) day 40. A reactive testing strategy was simulated whereby *N*_*sample*_ = 50 birds were sampled if no infection had previously been detected, with an increase to *N*_*sample*_ = 2000 following detection of infection.

For the second scenario to estimate the *T*_*s*_ with a known *R*_0_ we simulated sampling data using the rt-PCR test for four different introduction times: i) day 0, ii) day 7, iii) day 20, and iv) day 40 and for 4 different values of *R*_0_: 3, 5, 7 and 10. We assumed all birds would be tested for infection upon entry and exit to the barn (at the end of 8 weeks). We considered several intermediate sampling regimes to supplement this, where birds were additionally sampled once at week 4, three times at week 2, 4 and 6 and an additional 7 times between week 1-7.

### Parameter estimation

For each scenario we fitted the deterministic model to 100 different sets of stochastic observations. Model parameter values are given in Table 1.

When estimating *R*_0_ and *T*_*s*_ we assumed all other parameters were known. When investigating the effect of different sampling frequencies on the ability to estimate the *T*_*s*_ only we assumed the other parameters were known (including *R*_0_). For both scenarios parameters were estimated by maximising the product of the binomial likelihood across the time series of observed data using “optim” in R [25]. The likelihood of observing the sampling data at a given sampling time point *i* for a given set of model parameters is defined as:

15 $$\;\begin{array}{c} L\left(I_{\text{data}}|\theta \right)=\frac{N_{\text{sample}}!}{I_{\text{data}}!\left(N_{\text{sample}}-I_{\text{data}}\right)!}P_{i}^{I_{\text{data}}}{\left(1-P_{i}\right)}^{N_{\text{sample}}-I_{\text{data}}} \end{array}$$

where *N*_*sample*_ is the number of samples taken at that time point, *I*_*data*_ (the number of positive tests) and *P*, is the probability of a positive test (equation 12). To initialise the optimisation we performed a Markov Chain Monte Carlo (MCMC) search of the parameter space. For each chain we used 500 MCMC iterations to explore the likelihood surface when 1 parameter was estimated and increased to 5000 MCMC iterations for each chain when 2 parameters were estimated. Two Markov Chains with different starting conditions were simulated to ensure the parameter space was fully explored and to avoid the problem of chains becoming stuck in local optima.

We present the mean estimated *T*_*s*_ (and *R*_0_ where applicable) across 100 stochastic observations for each scenario and the mean 95% confidence intervals across the 100 observations for each scenario (Table 2 and Table 3). Profile likelihoods were estimated through the MCMC search of the parameter space, and confidence intervals were calculated from the profile likelihood, assuming the likelihood surface was approximately chi-squared distributed [26]. Where greater than one cluster was present (i.e. parameter sets with similar maximised likelihoods) both sets are presented. Confidence intervals were calculated for the clusters for which the maximum likelihood estimated (MLE) value was obtained and both are presented in Table 2.

**Table 2:** **Mean estimated** ***T***_***s***_ **and** ***R***_**0**_ **, from 100 stochastic realisations using an rt-PCR test**

Day of entry	***R***_***0***_10	***R***_***0***_7	***R***_***0***_5	***R***_***0***_3
0	-0.2 (-1.7, 1.2)	-0.6 (- 7.8, 1.8)	-0.8 (-2.8, 1.5)	-0.3 (-1.4, 0.5)
	10.5 (9.6, 12.0)	7.2 (6.9, 7.8)	5.0 (4.9, 5.8)	3.0 (2.9, 3.1)
7	7.1 (5.9, 8.5)	7.1 (5.8, 8.8)	7.0 (6.1, 7.8)	6.8 (5.6, 8.1)
	10.5 (9.0, 12.0)	6.9 (6.3, 8.1)	5.0 (4.8, 5.21)	2.9 (2.8, 3.1)
20	20.1 (18.3, 22.1)	20.0 (18.9, 21.0)	20.0 (18.1, 20.9)	19.4 (18.5, 21.9)
	10.5 (8.4, 13.8)	6.9 (6.3, 7.5)	4.8 (4,5, 5.1)	3.0 (2.9, 3.2)

Mean lower and upper 95% confidence intervals for each estimate are indicated in brackets. The upper estimate in each row refers to the estimate of *T*_*s*_ and the lower is the estimate of *R*_0_.

**Table 3:** **Mean estimated day of virus introduction into the barn, across 100 simulated testing observations**

Day of entry	***R***_***0***_	Weekly sampling	2 weekly sampling	4 weekly sampling
**Day 0**				
	10	0.0 (-0.6, 0.5)	0.1 (-0.7, 0.7)	0.0 (-2.3, 1.7)
	7	0.0 (-0.7, 0.6)	0.0 (-1.1, 0.9)	-0.1 (-1.7, 1.5)
	5	0.0 (-0.80, 0.9)	-0.3 (-1.8, 1.1)	-1.1 (-3.0, 1.1)
	3	0.1 (-1.4, 1.4)	0.0 (-2.3, 1.8)	-0.2 (-2.8, 2.5)
	3 2nd cluster	NA	NA	37.6 (26.7 - 40.0)
**Day 7**				
	10	7.0 (6.3, 7.5)	7.3 (6.0, 8.5)	7.2 (5.8, 8.6)
	7	7.0 (6.4, 7.6)	6.0 (4.9, 7.3)	2.6 (1.0, 4.5)
	5	7.0 (6.1, 7.9)	7.0 (5.9, 7.9)	7.2 (5.3, 8.8)
	3	6.9 (5.3, 8.3)	7.0 (5.5, 9.0)	6.6 (4.0, 9.5)
	3 2nd cluster	NA	NA	21.6 (17.1, 25.3)
**Day 20**				
	10	20.0 (19.4, 20.4)	20.2 (18.9, 21.5)	18.3 (16.0, 21.0)
	10 2nd cluster	NA	NA	46.6 (44.1, 50.0)
	7	20.0 (19.4, 20.9)	19.1 (17.8, 20.3)	18.0 (15.3, 21.3)
	7 2nd cluster	NA	NA	43.3 (40.5, 46.0)
	5	20.0 (18.9, 20.7)	20.0 (19.0, 20.9)	19.5 (18.1, 20.8)
	5 2nd cluster	NA	NA	34.5 (32.6, 36.6)
	3	20.1 (18.1, 22.2)	20.1 (17.7, 23.2)	19.7 (15.4, 23.4)
**Day 40**				
	10	40.0 (39.4, 40.7)	40.0 (39.9, 40.8)	36.0 (34.7, 37.1)
	10 2nd cluster	NA	NA	-9.56 (-10.0, -8.5)
	7	39.8 (38.1, 42.0)	40.0 (38.5, 41.5)	40.0 (38.5, 41.4)
	5	40.0 (36.7, 42.8)	39.7 (36.5, 42.8)	40.1 (36.7, 45.2)
	5 2nd cluster	NA	NA	13.3 (11.6, 15.0)
	3	37.6 (30.2, 43.7)	35.0 (28.2, 42.7)	33.5 (28.0, 40.8)

Mean lower and upper 95% confidence intervals for each estimate are indicated in brackets. The second optima that was observed with reduced sampling is referred to as 2nd cluster.

### Parameter values

The amount of virus shed, the durations of virus shedding and the infectious dose received upon an infectious challenge will vary and will be host specific, however we generalised values from the experimental data [27]. We assumed the latent period to be 2 days, based on a study which detected the shedding of H7N9 virus using oropharyngeal swabs in 10/11 chickens 2 days post inoculation (DPI) [27] (Pantin-Jackwood, personal communication), (although swabs were not taken between 0 and 2 DPI). We assumed the infectious period to be 6 days, based on the same study, as 7/8 birds were shedding detectable virus 8 DPI, however only 1/8 chickens was shedding virus 11 DPI [27] (Pantin-Jackwood, personal communication). The full experiment is detailed in Pantin-Jackwood *et al.*[27].

We assumed a fixed sensitivity and specificity of the rt-PCR test. The sensitivity estimate was based on a study where detection probabilities of 95% or greater were achieved for the HA(I) and NA(I) assays at RNA concentrations 7.0 and 7.8 copies per reaction, respectively. Although the confidence internals around the RNA concentrations to achieve this sensitivity were wide [20]. Specificity of the test was assumed to be 100%, as no non-specific cross reactivity of other oligonucleotides was reported. These values are similar to those published in other studies for the detection of H7 viruses [28, 29].

For the HI assay we assumed that the test had a very high specificity of 100% but a much lower sensitivity of 86%. At the time of writing no quantifiable data on the sensitivity and specificity of the H7N9 HI assay in avian hosts was available. We therefore used information on the sensitivity and specificity of HI assays for other avian subtypes [30] along with data available on the sensitivity and specificity of an antibody neutralisation assay [31] and an HI assay when testing human sera for antibodies to H7N9 infection [32].

### Sensitivity analysis

We conducted sensitivity analyses on 3 key parameters for which uncertainty surrounded the value used in the baseline model when estimating *T*_*s*_ in isolation, we performed sensitivity analysis on the following 3 parameters barn size, and the latent and infectious periods (results are presented in Additional file 1). It is likely barn size will vary across farms, so it was important to test our ability to estimate the day of virus introduction with variable barn sizes. The latent and infectious periods were based on observations from laboratory experiments, on a relatively small number of birds, and where heterogeneity between birds in the duration of virus shedding was observed [27], it was therefore important to explore variation in these parameters. Equally previous studies have shown that the duration of the latent period and infectious period can affect ones ability to detect virus in a barn [22, 33].

To investigate the model’s ability to estimate *T*_*s*_ with different barn sizes, we took an additional five different barn flock sizes, 500 birds, 2000 birds, 10,000 birds, 20,000 birds and 100,000 birds, while sampling *N*_*sample*_ = 5, *N*_*sample*_ = 20, *N*_*sample*_ = 100, *N*_*sample*_ = 200 and *N*_*sample*_ = 1000 birds from the flock at each sampling point, for each barn size respectively. To investigate how the misspecification of the duration of the latent period affected our ability to estimate the *T*_*s*_, we varied the value of the latent period used in the baseline model in the range 1-4 days. To investigate whether our estimation of *T*_*s*_ was sensitive to the duration of the infectious period, we tested values in the plausible range 3-12 days. All sensitivity analysis is presented within Additional file 1.

## Results

### Probability of detecting an outbreak at time of slaughter

**Figure 2:**
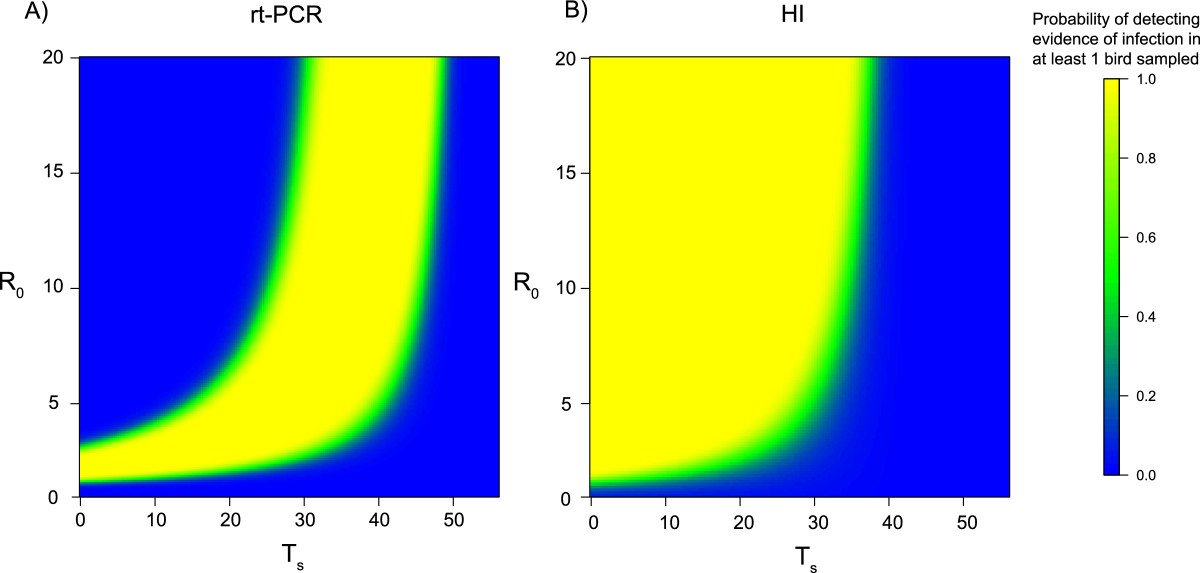
**The probability of detecting evidence of infection at time of slaughter with different basic reproduction numbers (** ***R***_**0**_ **) and days of virus introduction** ***T***_***s***_ **.** **A)** The probability of detecting at least 1 infected bird at time of slaughter (day 56) using an rt-PCR test, when 1% of the flock is sampled. **B)** The probability of detecting at least 1 infected bird at time of slaughter (day 56) using an HI assay, when 1% of the flock is sampled. For both panels, as the probability approaches 1 the greater the chance there is of detecting evidence of infection (yellow). As the probability approaches 0 there is no chance of detecting evidence of infection (blue).

We estimated the probability of detecting at least one infected bird at time of slaughter (56 days after birds enter the barn) using two different tests, rt-PCR and an HI assay. A non-linear relationship was observed between the probability of detecting evidence of an outbreak at the time of slaughter, *R*_0_ and the day of virus introduction (Figure 2) when using either the rt-PCR test or the HI assay.

The rt-PCR test had a high probability of detecting infected birds at the time of slaughter if *T*_*s*_ was between 20 and 40 days after birds enter the barn, across all values of *R*_0_ (Figure 2A). A high probability of detection for earlier *T*_*s*_ values was only apparent when *R*_0_ was less than 5, when the virus was likely to still be circulating at the time of slaughter. For the HI assay (Figure 2B) there was a high probability of detecting H7N9 antibodies at the time of slaughter across a range of *T*_*s*_ values (0 - 30 days), especially when *R*_0_ was high. However, if the *T*_*s*_ was 30 days or later, the probability of detecting infection was very close to 0, because of the 8 day period after infection required for birds to seroconvert. The probability of detecting at least one infected bird in the system when using either test was remarkably binary, with a very limited range of values giving a 50% probability of detecting evidence of infection. Overall the HI assay was predicted to detect evidence of an outbreak for a larger range of *R*_0_ values and introduction times than the rt-PCR test.

### Estimating *R*_0_and the day of virus introduction

**Figure 3:**
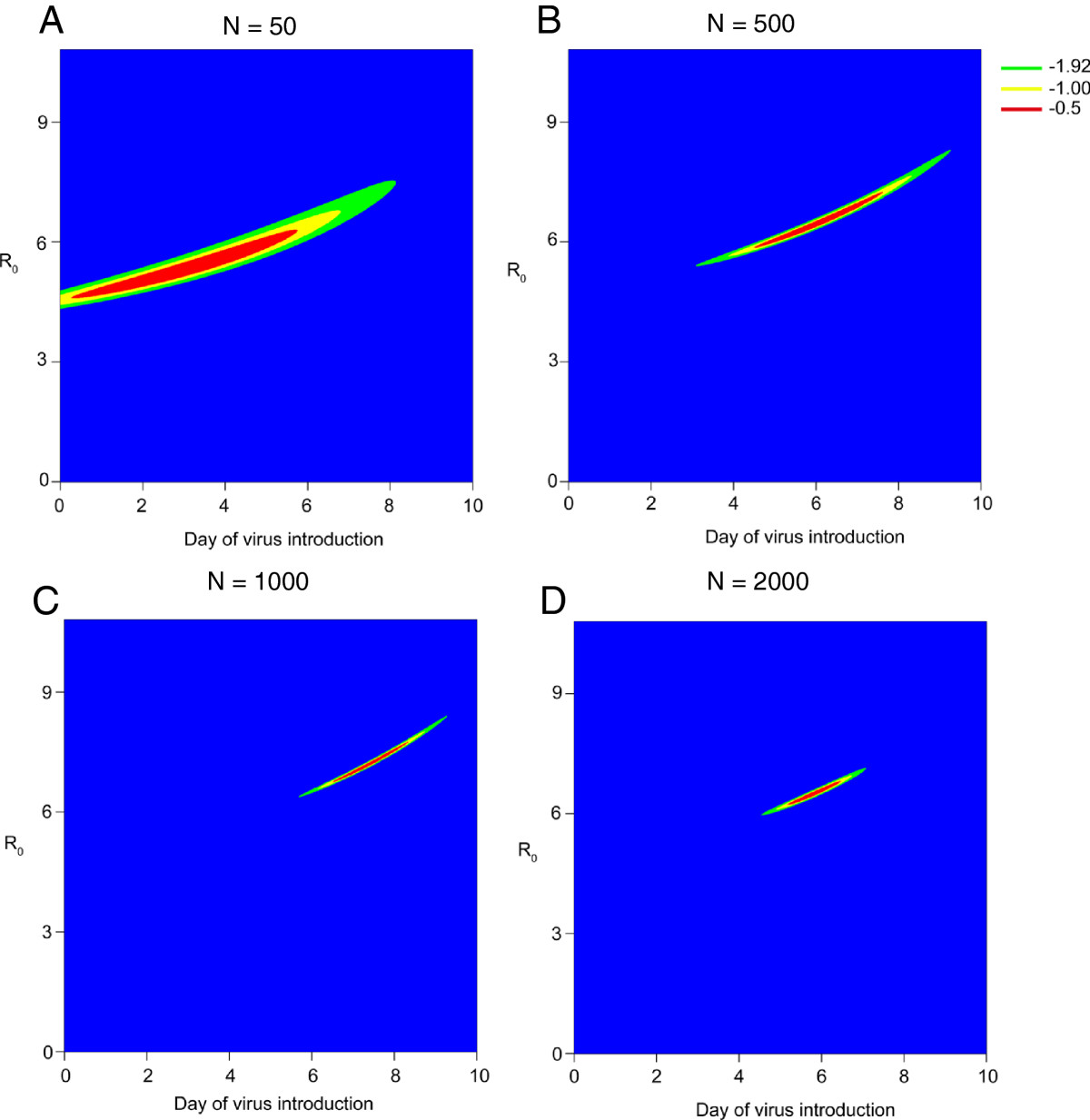
**The likelihood of identifying day of virus introduction (** ***T***_***s***_ **) and basic reproduction number (** ***R***_**0**_ **) simultaneously with and without varying degrees of reactive sampling.** **A-D** the contour of the log likelihood surface around the true values of *R*_0_ and *T*_*s*_. Reactive sampling of *N*_*sample*_ = 50, 500, 1000, and 2000 birds for A, B, C and D respectively. For illustration purposes an *R*_0_ = 7 and *T*_*s*_ = 7 were used. Different colours represent how close each estimate of the likelihood is to the maximum value. Blue indicates greater than 1.92 from the maximised true estimate, green indicates less than 1.92 likelihood point from the maximised true estimate (corresponding to the 95% confidence intervals), yellow indicates less than 1.0 likelihood point from the maximised true estimate and red indicates less than 0.5 points away from the maximised true estimate.

Birds within the barn were sampled and tested for infection using an rt-PCR test on a weekly basis to estimate *T*_*s*_ and *R*_0_, whilst assuming all other parameters were known (parameters are described in Table 1). In the absence of reactive sampling (an increase in the number of birds sampled at the next time point following the detection of infection), it was difficult to accurately obtain an estimate for *T*_*s*_ and *R*_0_. Here a wide range of different values of *T*_*s*_ gave comparable likelihoods for a fixed value of *R*_0_ (Figure 3A). We increased the number of birds sampled after the initial detection of infection in the following manner: *N*_*sample*_ = 50, *N*_*sample*_ = 500, *N*_*sample*_ = 1000 and *N*_*sample*_ = 2000, (Figure 3 A, B, C, D, respectively). The increase in number of birds sampled improved the accuracy of the estimates of *R*_0_ and *T*_*s*_ (Figure 3). Of the regimes tested the best accuracy arose from sampling 2,000 birds (40%) after detection of infection (Figure 3D). Anything lower resulted in highly variable estimates of *T*_*s*_ and *R*_0_ across different stochastic observations, therefore this was the sampling regime used for all estimates presented. We assumed that in the relatively rare event of such an outbreak resources would be extended to accommodate this sampling regime.

We present the mean *R*_0_ and *T*_*s*_ across 100 simulated observations for each scenario (Table 2). With weekly sampling, across the range of *R*_0_ values assessed here, we were able to simultaneously estimate both *R*_0_ and *T*_*s*_ (Table 2). In general all values of *R*_0_ were estimated accurately across the range assessed here. When considering *T*_*s*_ = 20, and *R*_0_ = 10, we estimated an *R*_0_ of 10.5 (9.6, 12.0), whilst, for an assumed *R*_0_ = 3, we estimated *R*_0_ 2.9 (2.8, 3.1) (Table 2). It was not possible to accurately estimate *R*_0_ and the *T*_*s*_ if the known day of virus introduction was day 40. This is because infection is very unlikely to be detected until day 56, as such we have only 1 informative data point on the presence of infection within the barn for the 8 week time series, thus it is not possible to make inference for the 2 parameters with only 1 data point. As such, a large number of different scenarios gave comparable likelihoods.

### Estimating the day of virus introduction under different sampling regimes and *R*_0_values

We subsequently estimated *T*_*s*_ on it’s own assuming known different values of *R*_0_ and variable sampling regimes. We considered a situation in the absence of reactive sampling, hence *N*_*sample*_ = 50 birds at each time point were sampled even after detection of infection.

We evaluated the quality of our estimate primarily in terms of accuracy, i.e. how close our estimate was to the true known value. In general *T*_*s*_ was accurately estimated across a range of *R*_0_ values and sampling regimes (Table 3). As expected, there was a reduction in the overall accuracy of the estimated *T*_*s*_ with each stepwise reduction in sampling frequency, with the most apparent reduction going from 2 weekly to 4 weekly sampling.

We present the mean estimated day of virus introduction across the 100 simulated observations for each scenario (Table 3). However, there was variation between the estimated day of virus introduction across the 100 data sets. We present the variation between the estimated day of virus introduction, for each scenario from each simulated dataset in Additional file 1. As would be expected, the most accurate estimates of *T*_*s*_ were obtained when a high frequency of sampling was used for a wide range of values of *R*_0_. At reduced sampling frequencies the precision of the estimate was greatly affected by the value of *R*_0_. For example, for *R*_0_ = 3, *T*_*s*_ = 40, and sampling conducted every 4 weeks, the estimate was less accurate compared to the true known value 33.5 (28.0, 40.8) (Table 3).

In some instances when intermediate sampling was conducted, only once during the 8 week cohort at week 4, different stochastic observations gave two different estimates of *T*_*s*_ with comparable maximised likelihoods, highlighting the presence of two different optima across the 100 stochastic observations. Results of both values are given in Table 3, where the other best estimated value, not close to the true value is referred to as the 2nd cluster in Table 3. In this sampling regime the estimation of *T*_*s*_ was strongly dependent on whether infection was detected on day 28 (2nd sampling point) or not, it was this observation process that resulted in different maximised likelihoods from different stochastic observations. The two different observed estimates arise because the model was unable to distinguish whether the epidemic was ending or taking off from 1 data point of number of infected’s at day 56. For example, if infection was introduced prior to day 28, but not observed when sampling was conducted on day 28 then *T*_*s*_ was overestimated, but if infection was observed on day 28 *T*_*s*_ was accurately estimated.

## Discussion

Outbreaks of LPAI are a threat to animal and human health, but are primarily asymptomatic in poultry. Therefore, if the threat to human health is considered high enough from strains such as novel avian H7N9, efficient surveillance strategies to detect virus and implement control measures may be required. We have simulated surveillance data from commercial poultry farms within the context of H7N9 outbreak surveillance and tried to understand the type of surveillance regime that would be required to help infer parameters and events of epidemiological importance.

In this study we used a mathematical model of transmission and testing to develop an understanding of the within flock dynamics during an outbreak of H7N9 in a single commercial poultry barn. We evaluated a number of different surveillance strategies that could be adopted on farms, and investigated how different surveillance regimes could infer parameters of epidemiological interest in the early stages of an outbreak, such as the day of introduction.

One surveillance strategy for monitoring the presence of LPAI outbreaks is to test birds at time of slaughter, which we assumed to be the end of the rearing period. In general we predicted that the HI assay would detect evidence of an outbreak for a wider range of introduction times and *R*_0_ values than the rt-PCR test. However the HI assay can only indicate that birds have experienced infection and cannot distinguish if birds are infected at time of slaughter. For outbreaks that commence 3-4 weeks after birds enter the barn, a higher probability of detecting evidence of an outbreak is expected using the rt-PCR test compared to the HI assay, as detection of live virus is possible very quickly after infection occurs. Importantly, if virus entered the barn during the last week; neither test would detect evidence of infection due to the slow emergence of antibodies for the HI assay and a very low probability of detecting infection using an rt-PCR test as the epidemic would have only just begun in the barn. Our analysis assumes that after rearing, birds would be transported to a slaughterhouse. There may be scenarios where birds are transported to another facility prior to slaughter. If they are tested for infection at this second facility our results suggest that using an HI assay would be a more appropriate than an rt-PCR test, as the overall time period for which it would be possible to detect evidence of infection would be higher. Equally, although most large facilities will have more than a single barn, our results are still applicable in that they are designed to detect the virus in the first affected barn. Once a novel strain is detected in a single barn on a large facility, it is likely that either frequent testing or pre-emptive culling would be started rapidly in other barns as resources would not be constrained in the same way as facilities with no history of infection.

The threat and transmission potential of H7N9 in commercial chickens remains largely unknown, and the within flock *R*_0_ remains unquantified. A number of previous studies have used different methodologies to quantify *R*_0_ and the transmission rate *β* from different outbreak settings [33–35]. Within barn estimates have been highly variable across settings and viral subtypes (*R*_0_ = 2.26 to 5.50) [33–35], therefore it was important to consider a wide range of values. The *R*_0_ value is also likely to vary between countries and farms due to different farming practices. Therefore we examined our ability to estimate the *T*_*s*_, whilst also estimating *R*_0_, which might be a priority during the initial stages of a national outbreak. It was not possible to accurately estimate the two parameters simultaneously when sampling only *N*_*sample*_ = 50 (Figure 3A) (1%) of birds at each time point as the likelihood surface was relatively flat. We found it was necessary to sample at least *N*_*sample*_ = 2000 (40%) birds after the detection of infection to accurately estimate *R*_0_ and *T*_*s*_. We saw the most accurate parameter estimation was performed when *T*_*s*_ was either 7 or 20 days, with the model performing less accurately for day 0. It was not possible to estimate *T*_*s*_ if virus was introduced on day 40.

In situations where *R*_0_ is known it would be possible with regular testing to estimate *T*_*s*_. We investigated this approach using the rt-PCR test, over different frequencies of testing where the *R*_0_ value was known. We found that if sampling *N*_*sample*_ = 50 birds at each sampling time point, testing for infection on a weekly basis provided the most precise and accurate estimate of *T*_*s*_. We observed a slight reduction in the accuracy of our estimate as the value of *R*_0_ decreased. This is likely due to epidemics with a lower *R*_0_ exhibiting a slower take off which in turn lowers the probability that at a particular point in time infection will be observed as a lower number of overall birds will be infected.

Regular sampling regimes with 4 or more samples in the 8-week period did much better than 3 or fewer samples. We saw that with 4 weekly sampling (3 samples) the model was very sensitive to observations taken on day 28. If infection was not detected then a different global optimum was identified that did not correspond to the true known day of introduction. The presence of two global optima under different stochastic observations of the infection dynamics resulted in uncertainty in the estimated time of introduction. The comparison of entry times between 1 and 2 weekly sampling showed little difference in the point estimates between the two regimes, however 2 weekly sample provided a wider range on the 95% confidence intervals.

Through our sensitivity analysis we have shown that the model estimation is robust to changes in flock size, as well as to the misspecification of the duration of the latent period. However the model was sensitive to changes in the infectious period especially when the infectious period was greatly extended. It has been reported that most birds infected with AI shed virus for around 7 days [36–38], so the upper thresholds explored here may be considered unrealistic.

The differential susceptibility of avian hosts to different influenza strains has been widely described. Turkeys are more susceptible to H7N2 infection than chickens [39], while Muscovy ducks have a higher susceptibility to HPAI H5N1 infection than mallards [40]. Here we focus on chickens because human infections have primarily been associated with chickens from live animal markets [41, 42]. However, it is known that other avian species have been identified as infected within live birds markets in China. Despite possible differences in susceptibility or durations of infectiousness of other avian hosts compared to chickens the same model structure and methodology could still be applied.

## Conclusions

We have shown that an unfeasibly large number of birds would need to be sampled to obtain an accurate estimate of *R*_0_ and *T*_*s*_ simultaneously. However, a once off significant investment to sample such a high number of birds would be of high utility and justifiable in the early stage of an outbreak, particularly if it was suspected or known that multiple premises were infected. Equally, the high number of birds that would need to be sampled to obtain a precise and accurate estimate of the 2 parameters is likely to reduce as *R*_0_ increases, however in a situation where *R*_0_ is unknown, caution should be taken. We have also shown that significantly fewer resources would be required to estimate *T*_*s*_ alone, where *N*_*sample*_ = 50 on a weekly or 2 weekly basis during an 8 week cohort, using an rt-PCR test would be enough to provide a good estimate of *T*_*s*_.

The inference of *R*_0_ during the early stages of an outbreak is of high utility in the early stages of an outbreak as it enables control and infection containment strategies to be implemented effectively, and outbreak responses to be co-ordinated. For example, quantifying a value for *R*_0_ can help understand the number of birds that would need to be vaccinated, how quickly a virus can spread through a flock, and if culling would be necessary. Equally, estimating *T*_*s*_ with or without *R*_0_ can dramatically reduce the resources required to identify the source of infection and may help gain understanding as to whether additional premises have been infected, and can prevent onwards transmission. As such, we feel our method and its findings are of high utility to help guide surveillance to improve the estimation of epidemiological parameters from outbreak data.

## Electronic supplementary material

Additional file 1: **Variation in the estimated day of virus introduction for the single parameter model and sensitivity analysis for the estimation of** ***T***_***s***_ **.**(PDF 577 KB)

Below are the links to the authors’ original submitted files for images.

Authors’ original file for figure 1

Authors’ original file for figure 2

Authors’ original file for figure 3

## Abbreviations

AI: Avian influenza
LPAI: Low pathogenic avian influenza
HPAI: High pathogenic avian influenza
rt-PCR: Real-time polymerase chain reaction
HI assay: Hemagglutinin inhibition assay
*T*_*s*_: Day of virus introduction
*R*_0_: Basic reproduction number
DPI: Days post inoculation.
