# Supplementary material for: Surveillance of low pathogenic novel H7N9 avian influenza in commercial poultry barns: detection of outbreaks and estimation of virus introduction time
Source: BMC Infect Dis. 2014 Aug 1;14:427. doi: 10.1186/1471-2334-14-427 (PMC4129106; doi:10.1186/1471-2334-14-427)
Supplement: Supplementary file 1 — Additional file 1: Variation in the estimated day of virus introduction for the single parameter model and sensitivity analysis for the estimation of T s .(PDF 577 KB) [file 12879_2014_3725_MOESM1_ESM.pdf]

Additional File 1

Variation in the estimated day of virus introduction for the single parameter model

We illustrate how much variation these was across the 100 stochastic observations for each scenario. We present the distribution of estimates for each sampling regime, day of virus introduction and value of R_0. We did observe variation in the estimated day of virus introduction within each scenario, the variation is shown in Additional file 1: Figures S1- S4, each figure represents a different day of virus introduction. Overall the best estimates were obtained through weekly sampling.

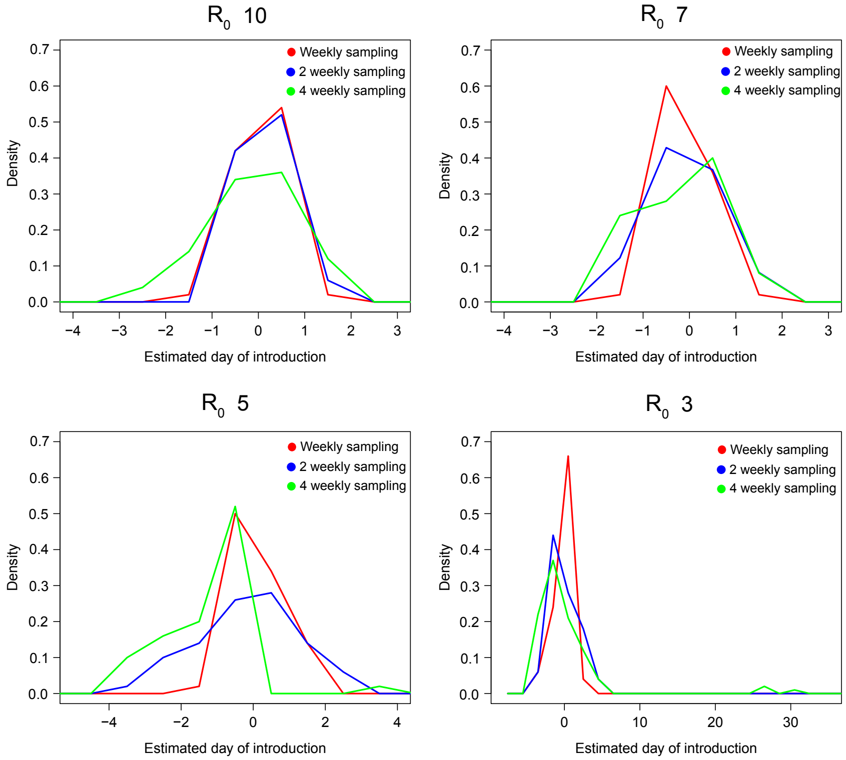

Figure S1: **Distribution of estimated day of virus introduction for day 0 under a range of R_0 values and sampling regimes.** Different sampling regimes for each value of R_0 are indicated by colour. Weekly sampling in red, 2 weekly sampling in blue and 4 weekly sampling in green.

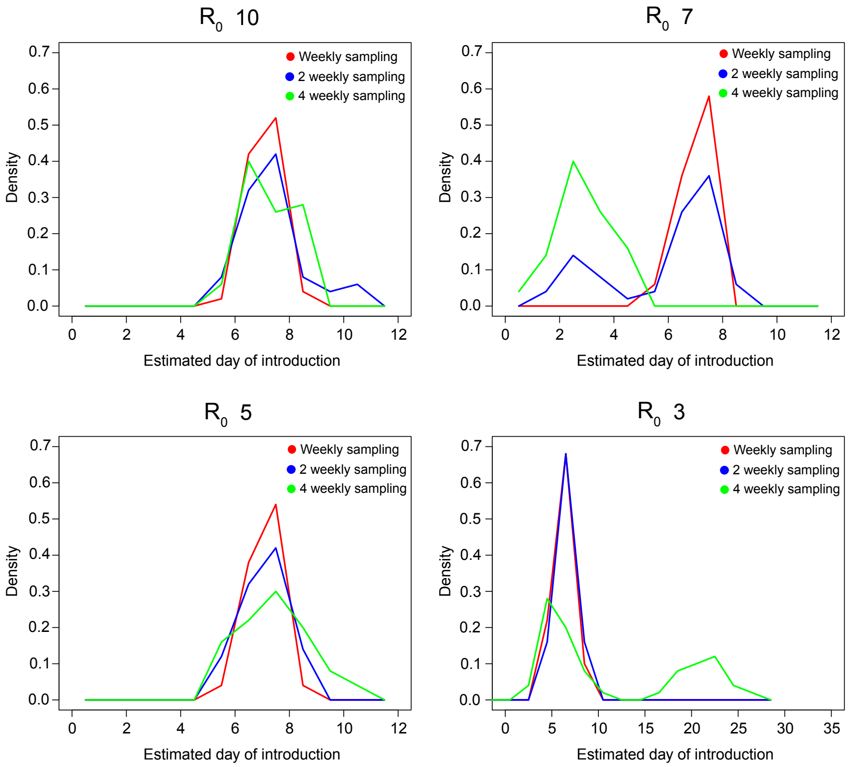

Figure S2: **Distribution of estimated day of virus introduction for day 7 under a range of R_0 values and sampling regimes.** Different sampling regimes for each value of R_0 are indicated by colour. Weekly sampling in red, 2 weekly sampling in blue and 4 weekly sampling in green.

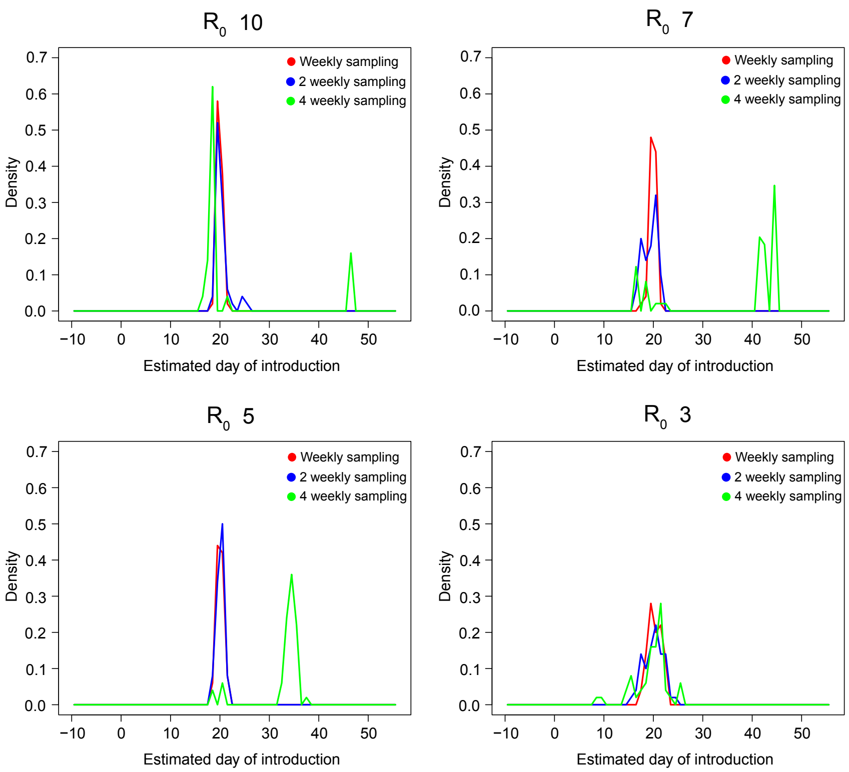

Figure S3: **Distribution of estimated day of virus introduction for day 20 under a range of R_0 values and sampling regimes.** Different sampling regimes for each value of R_0 are indicated by colour. Weekly sampling in red, 2 weekly sampling in blue and 4 weekly sampling in green.

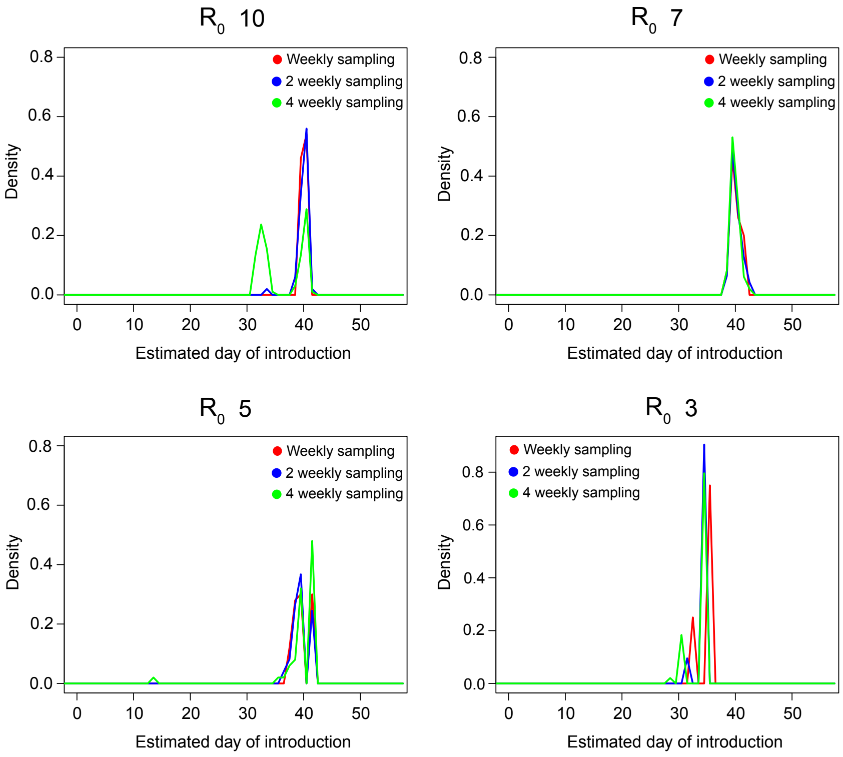

Figure S4: **Distribution of estimated T_s for day 40 under a range of R_0 values and sampling regimes.** Different sampling regimes for each value of R_0 are indicated by colour. Weekly sampling in red, 2 weekly sampling in blue and 4 weekly sampling in green.

Sensitivity analysis

We assessed our ability to estimate T_s under variable barn sizes, latent and infectious periods, whilst also varying R and the frequency of sampling. Results are indicated in Additional file 1: Table S1, S2 and S3 respectively for each parameter.

The point estimate of T_s appeared reasonably insensitive to changes in flock size within the barn across the values of R_0 and range of T_s values assessed here (Table S1). Although when considering a flock size of 500 with less frequent sampling estimates were less close to the true value than the other flock sizes tested here. Suggesting that in general changes in the flock size do not have a serious effect on our ability to estimate the T_s compared to the baseline model, however if being used on much smaller flock sizes with a reduced sampling frequency caution should be applied when interpreting the results.

For weekly sampling T_s was accurately estimated across the variable range of latent periods (Table S2). For low values of R_0 and low frequency sampling regimes we saw poorer estimates of T_s compared to the baseline model. For example, with a 4 day latent period, with 4 weekly sampling and $R_0 = 3$, T_s was estimated to be 25, whereas the baseline model estimated 33 days, suggesting that the model may be sensitive to longer latent periods at lower sampling frequencies. We saw that with a 1 day latent period and a high R_0, the epidemic took off very quickly and also finished very quickly, resulting in a slight overestimation of T_s. As with the baseline model, instances in which 4 weekly sampling was undertaken resulted in two very different point estimates, as a result of different optima for different stochastic observations.

For weekly sampling the model appeared insensitive to a reduction or increase in the duration of the latent period (Table S3). In general for 2 weekly sampling we saw comparable estimates of the T_s to the baseline model, except for when R_0 was low and the virus was introduced on day 40. As with the baseline model for 4 weekly sampling we saw that sometimes two different optima were detected across the 100 stochastic observations. We see an anomaly in the data when the latent period was 3 days, R_0 was 10 and virus was introduced on day 40: T_s was consistently under estimated. Likely to be because with higher values of R_0 the model was unable to distinguish whether the epidemic

is taking off or coming down when sampling was infrequent and infected birds were only detected on day 56.

Table S1: Mean estimated day of virus introduction varying barn size, across 100 simulated testing observations. We consider a flock of 500, 2000, 10,000, 20,000 and 100,000 birds.

**Day of entry**	R_0	**Weekly sampling** 0.5, 2, 10, 20, 100 (thousand) birds	**2 weekly sampling** 0.5, 2, 10, 20, 100	**4 weekly sampling** 0.5, 2, 10, 20, 100
**Day 0**				
	10	-0.2, 0.0, 0.0, 0.0, 0.0	-2.0, -1.5, 0.0, 0.0, 0.0	4.5, -1.0 , 0.0, 0.0, 0.0
	7	-0.2, 0.0, 0.0, 0.0, 0.0	-4.7, 0.0, 0.0, 0.0, 0.1	3.2, 0.0, 0.0, 0.1, 0.0
	5	-0.2, 0.0, 0.0, 0.0, 0.0	-0.1, -0.2,-0.1, 0.0, 0.0	0.9, -0.4, 0.0, 0.0, 0.0
	3	-0.1,-0.1, 0.0, 0.0, 0.0	-1.5, 0.0, -0.2, 0.0, 0.1	-0.2, 0.3, 0.5, 0.3, 1.3
**Day 7**				
	10	6.8, 7.0, 7.0, 7.0, 7.0	7.7, 7.0, 7.2, 6.6, 7.0	7.4, 6.9, 7.0, 5.7, -0.94
	7	7.0, 6.9, 7.0, 7.0, 7.0	7.2, 7.1, 7.0, 7.0, 7.6	7.0, 6.4, 6.8, 7.0, 7.0
	7 2nd cluster	NA	-1.0, 32.3, -3.4, NA	
	5	6.3,6.9, 6.9, 7.0, 7.0	6.1, 7.1, 7.0, 7.1, 6.9	6.0, 7.0, 6.9, 7.0, 6.9
	5 2nd cluster	NA	0.8, NA ,NA ,NA ,NA	1.3 ,-1.2, NA, NA, NA
	3	6.5, 7.0, 7.0, 7.0, 7.0	8.1, 6.8, 7.1, 7.1, 6.9	8.4, 6.7, 10.8, 8.7, 6.9
	3 2nd cluster	NA	NA	-2.4, NA, NA, NA, NA
**Day 20**				
	10	19.9, 20.0, 20.0, 20.0, 20.0	19.9, 20.6, 20.7, 20.1, 20.4	6.1, 21.0, 19.2, 19.9, 19.9
	10 2nd cluster	NA	NA	NA, NA, 35.1, 46.2, 40.3
	7	20.1, 20.0, 20.0, 20.0, 20.0	19.9, 19.9, 19.8, 19.9, 20.0	3.3, 20.3, 19.6, 19.9, 20.0
	7 2nd cluster	NA	NA	NA, NA, 41.5, 38.4, 31.6
	5	19.8, 19.8, 20.0, 20.0, 20.0	19.4, 18.5, 20.0, 20.0, 20.0	0.51, 19.2, 19.88, 27.1, 19.9
	5 2nd cluster	NA	NA	41.32, NA, 31.4, NA, NA
	3	20.6, 20.1, 20.1, 20.1, 20.0	22.0, 20.4, 20.1, 19.9, 20.0	28.7, 21.0, 19.5, 20.2, 20.0
**Day 40**				
	10	40.8, 40.1, 39.6, 38.8, 38.5	41.7, 39.6, 40.0, 39.8, 40.0	40.5, 39.9, 38.8, 39.7, 40.0
	7	39.7, 40.1, 40.1, 40.2, 39.1	40.0, 40.0, 40.2, 40.1, 39.7	37.6, 39.4, 40.0, 39.7, 40.2
	5	39.3, 37.8, 40.2, 40.2, 37.7	39.0, 40.0, 39.9, 40.1, 39.2	36.4, 40.0, 40.0, 39.9, 39.9
	5 2nd cluster	NA	NA	NA, 12.1, NA, NA
	3	34.6, 36.7, 35.6, 37.4, 36.8	31.2, 34.6, 35.6, 35.2, 36.0	30.7, 32.0, 35.2, 34.5, 34.5

Table S2: Mean estimated day of virus introduction with variable latent periods, across 100 simulated testing observations.

**Day of entry**	R_0	**Weekly sampling**<br>1, 3, 4 days	**2 weekly sampling**<br>1, 3, 4 days	**4 weekly sampling**<br>1, 3, 4 days
**Day 0**				
	10	0.0, 0.0, 0.1	-0.2, 0.0, 0.0	2.6, 0.0, -0.7
	7	0.0, 0.0, 0.0	-0.5, 0.0, 0.1	0.2, -0.6, 0.0
	5	0.1, 0.1, -0.1	0.0, 0.1, 0.0	0.0, 0.0, 0.6
	3	-0.1, 0.0, 0.3	-0.1, 0.0, 0.0	-5.0, 2.9, -0.8
**Day 7**				
	10	7.0, 7.0, 7.0	7.8, 6.7, 7.0	6.9 ,6.7, 7.1
	7	7.0, 7.0, 7.1	7.0, 7.1, 7.2	7.1, 7.1, 7.7
	5	7.0, 7.0, 7.0	6.5, 7.0, 7.0	5.8, 7.8, 6.5
	5 2nd cluster	NA	NA	NA, NA, 24.2
	3	6.8, 7.0, 7.2	7.1, 7.1, 7.5	7.1, 7.8, 7.3
	3 2nd cluster	NA	NA	NA, NA, -6.6
**Day 20**				
	10	20.1, 20.0, 20.0	20.2, 19.1, 20.0	19.5, 18.1, 20.0
	10 2nd cluster	NA	NA	35.7, 42.7, 34.7
	7	20.0, 20.0, 20.0	21.7, 19.9, 20.7	19.7, 19.7, 24.5
	7 2nd cluster	NA	NA	0.86, 34.5, NA
	5	20.0, 20.1, 20.0	20.2, 20.5, 20.0	21.9, 22.9, 19.8
	3	20.1, 20.5, 20.5	20.4, 20.3, 20.2	27.1, 20.1, 20.3
	3 2nd cluster	NA	NA	NA, - 3.8, NA
**Day 40**				
	10	40.1, 39.2, 39.8	43.4, 39.3, 39.9	40.1, 40.1, 40.2
	10 2nd cluster	NA	NA	29.1, NA, NA
	7	39.9, 39.0, 38.4	40.0, 39.9, 38.2	38.6, 39.9, 38.0
	7 2nd cluster	NA	NA	-8.2, NA, 8.7
	5	40.0, 38.6, 37.7	39.2, 37.9, 35.8	38.5, 37.7, 34.6
	3	39.5, 37.7, 34.4	38.8, 29.9, 31.0	38.6, 40.1, 25.0

Table S3: Mean estimated day of virus introduction with variable infectious periods, across 100 simulated testing observations.

Day of entry	R_0	Weekly sampling<br>3, 9, 12 days	2 weekly sampling<br>3, 9, 12 days	4 weekly sampling<br>3, 9, 12 days
**Day 0**				
	10	0.1, 0.0, 0.0	-0.2, 0.0, 0.1	-0.1, -0.1, -0.3
	7	0.0, 0.0, 0.1	-0.1, -0.2, 0.0	1.8, -1.6, 0.0
	5	0.2, 0.0, 0.0	0.4, 0.0, 0.0	0.0, 0.0, -0.1
	3	0.0, 0.0, 0.0	-0.2, 0.4, -0.4	0.0, 2.4, 0.0
**Day 7**				
	10	6.8, 7.0, 7.1	6.4, 6.8, 7.1	6.9 ,7.3, 7.1
	10 2nd cluster	NA	19.9, NA, NA	20.2, 20.3, NA
	7	7.1, 7.0, 7.0	7.0, 7.0, 7.2	6.8, 7.1, 6.8
	7 2nd cluster	NA	NA	15.4, -9.8, NA
	5	7.1, 7.0, 7.0	8.7, 6.9, 7.1	7.4, 8.3, 6.5
	5 2nd cluster	NA	NA	NA, NA, 18.8
	3	6.8, 6.9, 7.1	7.0, 7.0, 7.3	7.3, 6.1, 7.1
**Day 20**				
	10	20.2, 20.0, 20.0	20.8, 19.5, 20.1	19.9 ,18.8, 20.0
	10 2nd cluster	NA	NA	7.6, 40.9, NA
	7	20.0, 20.0, 20.0	24.9, 20.0, 20.4	18.7, 19.4, 22.9
	7 2nd cluster	NA	NA	3.6, 32.9, NA
	5	20.0, 20.0, 20.0	22.7, 20.6, 19.9	19.4, 21.4, 19.6
	5 2nd cluster	NA	NA	0.0, NA, NA
	3	19.8, 21.0, 20.5	20.0, 20.9, 20.2	33.3, 20.1, 19.7
**Day 40**				
	10	40.7, 38.7, 39.2	43.7, 40.2, 40.2	-0.6, 40.2, 39.8
	7	39.8, 40.1, 39.7	40.0, 39.6, 38.4	38.6, 39.9, 38.3
	5	40.0, 38.6, 38.5	39.9, 37.9, 35.9	39.9, 37.7, 35.1
	3	39.3, 30.5, 33.6	38.7, 32.8, 29.9	38.8, 28.9, 23.4
