# Supplementary figures and images for: Surveillance of low pathogenic novel H7N9 avian influenza in commercial poultry barns: detection of outbreaks and estimation of virus introduction time
Source: BMC Infect Dis. 2014 Aug 1;14:427. doi: 10.1186/1471-2334-14-427 (PMC4129106; doi:10.1186/1471-2334-14-427)

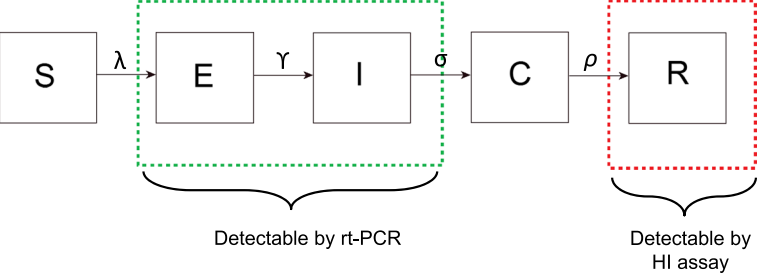

Supplement: Supplementary file 2 — Authors’ original file for figure 1 [file 12879_2014_3725_MOESM2_ESM.pdf]

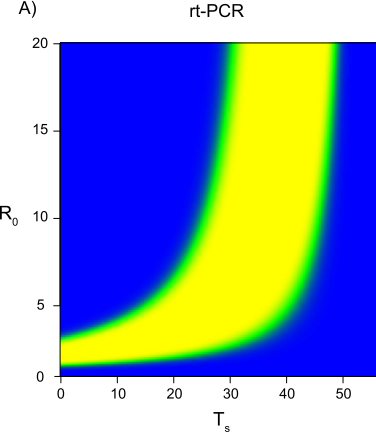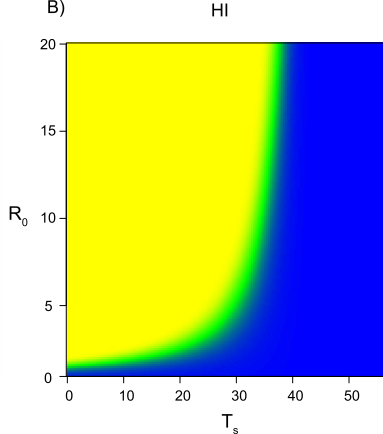

Probability of detecting  
evidence of infection in  
at least 1 bird sampled

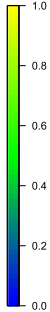

Supplement: Supplementary file 3 — Authors’ original file for figure 2 [file 12879_2014_3725_MOESM3_ESM.pdf]

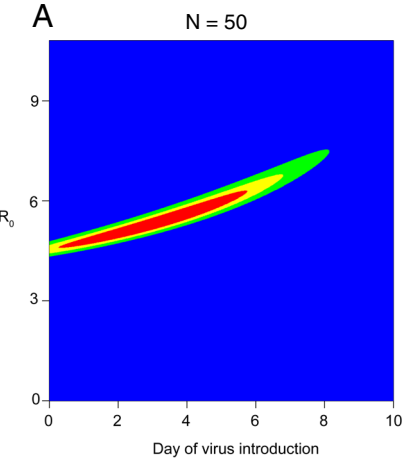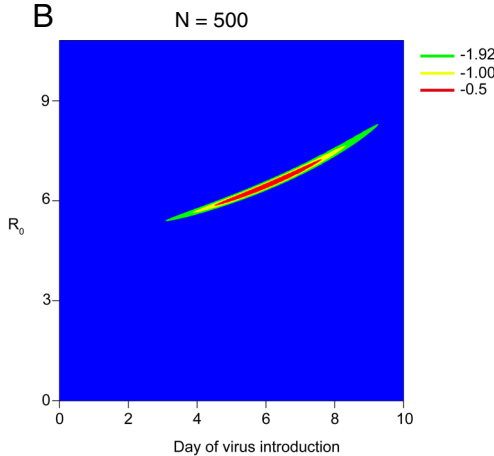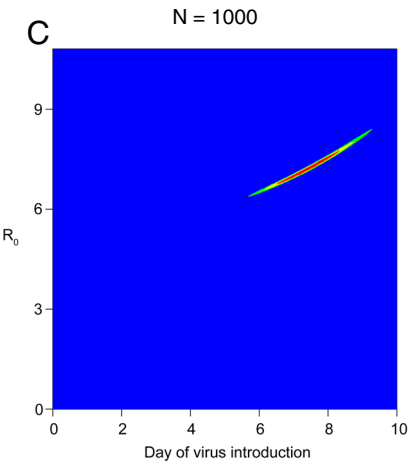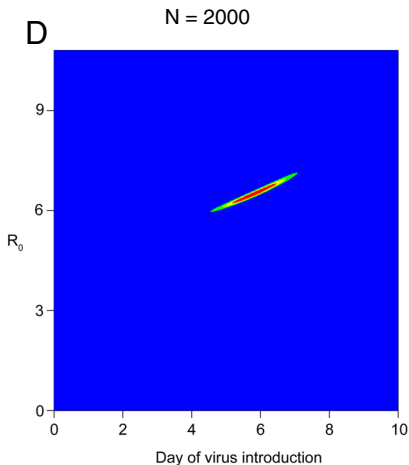

Supplement: Supplementary file 4 — Authors’ original file for figure 3 [file 12879_2014_3725_MOESM4_ESM.pdf]
